# Highlights of the second ISCB Student Council Symposium in Africa, 2017

**DOI:** 10.12688/f1000research.13463.1

**Published:** 2017-12-28

**Authors:** Candice N. Rafael, Efejiro Ashano, Yumna Moosa, Sayane Shome, Dan DeBlasio

**Affiliations:** 1 Research Unit in Bioinformatics, Department of Biochemistry and Molecular Biology, Rhodes University, Grahamstown, South Africa; 2 Immunovirology and Vaccine Development Laboratory, Medical Biotech Department, National Biotechnology Development Agency, Abuja, Nigeria; 3 KwaZulu-Natal Research and Innovation Sequencing Platform (KRISP), University of KwaZulu Natal, Durban, South Africa; 4 Bioinformatics and Computational Biology Program, Iowa State University, Ames, IA, USA; 5 Computational Biology Department, Carnegie Mellon University, Pittsburgh, PA, USA

**Keywords:** ISCB Student Council, Student Council Symposium, conferences, meetings, Africa

## Abstract

Student Council Symposiums (SCSs) have been found to be very useful for students and young researchers. This is especially true given that the events are held directly before large international conferences, giving attendees a chance to gain exposure and have a warm up to the social nuances involved in attending such a meeting. This was the second SCS held in Africa in conjunction with the International Society for Computational Biology (ISCB) and the African Society for Bioinformatics and Computational Biology’s (ASBCB) biennial meeting. This symposium was organised by students within the society inside Africa and was held on the 10 ^th^ of October 2017 in Entebbe, Uganda.

## Introduction

Bioinformatics is a rising and developing field, this is especially true within Africa, and it is the International Society for Computational Biology (ISCB) Student Council’s mission to help facilitate the development of young bioinformaticians throughout the world. To this end the Student Council sponsored the 2nd African ISCB Student Council Symposium (SCS), which was held alongside the ISCB-Africa African Society for Bioinformatics and Computational Biology (ASBCB) 2017 conference. This was the fifth joint meeting of ISCB and ASBCB, and the sixth conference of the ASBCB focusing on Bioinformatics of African pathogens, hosts and vectors including the showcasing of the progress and breakthroughs made through the H3Africa and H3ABionet programs ^a^.

Student Council Symposia aim to attract young scientists within the field to a meeting where they are given opportunities to showcase their work and get feedback in a slightly more relaxed environment than at a large international conference. All of the SCS events are organised by students with a focus on highlighting the work undertaken by students and they provide a unique opportunity to both the participants and organizers. One major benefit for participants in the symposium is the ability to network with future colleagues who have similar interests and hail from locations across the continent as well as gain exposure to a broad range of research taking place internationally they may not otherwise encounter. The benefit of having the SCS meetings directly before the main ISCB conferences is to allow those who have not attended a conference before to get a gentle introduction surrounded by only other students. The organizers get the opportunity to see a conference from the managerial perspective. From conception, to abstract submission and review, to day-of organizing and session chairing; these are activities that they may not be able to participate in until much later in their career.

The SCS series has been a major activity of the ISCB Student Council (ISCB-SC) since its conception in 2003. The SCS meetings have been held annually in conjunction with the Intelligent Systems for Molecular Biology (ISMB) conference since 2005. In 2010 the European SCS series started as the first regional SCS and precedes the European Conference on Computational Biology (ECCB) in years when ISMB and ECCB are not co-located. Do to the successes of these two annual/biennial meetings SCS-Africa (SCS-A) and SCS-Latin America were established in 2015 to be co-located with the two regional flagship ISCB conferences (ISCB-Africa ASBCB and ISCB-LA).

SCS-A is one of the main focuses of the four Regional Students Groups (RSGs) of Africa. The RSGs, and the ISCB-SC as a whole, focus their efforts on facilitating and supporting the development of the next generation of computational biologists ^1– 3^. The ISCB-Africa ASBCB conference takes place every two years with the SCS-A set to occur alongside it. The first SCS-A meeting which took place in 2015 in Tanzania and was a great success ^4^.

This years installation of SCS-Africa was held on the 10 ^th^ of October 2017 in Entebbe, Uganda directly before ISCB-Africa ASBCB 2017. Detailed program information can be seen online ^b^.

## Format of the meeting

SCS-Africa 2017 was a half-day event that began with a welcome from the conference organisers followed by a keynote address, icebreaker and five student presentations. The event was concluded by thanking participants, sponsors, organisers, and speakers followed by a group poster session.

The symposium was attended by 44 students from 9 different countries across the continent and included participants at all levels of expertise from undergraduate through to post doctorate and young researchers. These students were from a variety of backgrounds including: immunology, virology, biology, genetics, physics, mathematics, statistics, software development, bioinformatics and computational biology.

The student speakers submitted abstracts a few weeks before the meeting and these submissions were reviewed by members of the ISCB-SC across the globe. SC has a broad network of students within the field that gave feedback on the abstracts in order for the organizers to ultimately make a decision on their acceptance and order. The final abstracts are available online ^c^ and each of the talks is summarized below.

### Welcome address

SCS-Africa began with a welcome address introducing the ISCB-SC’s structure, function, and purpose, by the current vice-chair for African RSGs in Student Council and previous president of the RSG-South Africa, Candice Rafael. The program overview was given and participation in the day was encouraged.

### Keynote

Dr. Segun Fatumo, the current vice-president of the ASBCB and founder and pioneer president of RSG-Africa, was the keynote for the SCS-Africa. His talk commenced with showing the progress of the Student Council and RSGs within Africa from the first ISCB-Africa ASBCB in 2007 to now, and highlighted the importance of being involved in Student Council in the context of a scientific or academic career. This portion included examples from his own experience and career. Dr. Fatumo then went on to highlight some of his own research on cardiometabolic risk factors and its metabolic consequences of hypertension, type-2 diabetes, cardiovascular disease, and other co-morbidities in relation to African populations.

### Icebreaker

A brief icebreaker was held to encourage students to move from their comfort zone and interact with each other. This was held in the format of “Scientific Speed Dating” as used previously at other SCSs ^5^. Students were encouraged to move around the room and start conversations with other attendees they did not travel with or did not know previous to the SCS. After 3 minutes a timer sounded signaling the students to move to new areas and start over with a different attendee. This was carried on for 5 rounds. Students were then encouraged to continue these conversations over tea and lunch breaks.

### Student presentations

Gerald Mboowa, a PhD student from Uganda, gave the first talk titled “Host genetic polymorphisms associated with malaria resistance in HIV-infected children: a retrospective study from Sub-Saharan Africa”. Gerald took us through his study, wherein he proposed to identify novel host genetic polymorphisms associated with malaria resistance/susceptibility in HIV-infected children in sub-Saharan Africa

Olaitan Awe, a PhD student from Nigeria, presented work from his PhD titled “ *In-silico* identification of protein-coding and non-coding regions in next-generation technology transcriptome sequence data: a machine learning approach”. He highlighted a computational model, which was developed using machine learning techniques to identify protein-coding and non-coding RNA transcripts, including partial-length RNA transcripts, and the resulting model gave a very good statistical generalization performance of 96.5% in each of prediction accuracy, F1-score, specificity and sensitivity.

Oladipo Kolawole a PhD student from Nigeria, delivered a talk on his PhD research “Comparative structural analysis of 3-D predicted matrix protein of influenza a H1N2/Ibadan/2014 and H5N1/Ogbomoso/2014 in Nigeria”. Oladipo showed the compared predicted 3-D protein structures of the obtained Influenza A virus Matrix protein has provided additional insights into the strain-specific nature which has revealed the various variations. He concluded that it is likely that the structural differences in their protein structure accounts for their pathogenicity.

Marion Amujal, a PhD student from Uganda, presented her work on “Whole genome assembly and functional significance of genetic variants of *Mycobacteria tuberculosis* isolated from Kampala, Uganda”. Marion presented her work on the *Mycobacterium tuberculosis* complex (MTC) and specifically the Uganda genotype that has now been found to be prevalent in the East African region. This genotype has been reported to have a higher infectious rate and lower drug resistance than other genotypes, however the mechanism underlying this is not well understood. Her work is therefore focused around understanding this genotype in terms of genome structures and gene repertoires.

Usman Adeyemi Lamidi, a PhD student from Nigeria, ended the student talks with his work entitled “Motif discovery in DNA sequences using an improved Gibbs sampling algorithm”. Here Usman present an *Improved Gibbs* (IGibbs) sampling algorithm on Breast Cancer human disease DNA sequences that involves altering the processes to obtain a reduced runtime and also achieve an accurate satisfactory motif result.

### Poster session

All students who attended were encouraged to bring a poster to share their work. These were displayed on poster boards and all attendees were given a chance to interact with the presenters. Students were able to present their work, leading to not only scientific feedback, but also useful experience that aided in presenting their work in the main conference.

## Concluding remarks

Events such as this are a useful and vital platform for students in their development as young scientists. This can also form an important opportunity for networking and collaboration building among students from a wide and varied background as well as from distant locations across the continent. The inclusion of the icebreaker was very well received and students enjoyed the opportunity to interact. Since the Student Council Symposia are organised by students, for students, it is easily adaptable to the needs and preferences of those organizing and participating. Through our experience we have provided feedback to the next set of symposia organizers, such as the inclusion of more time for organized open discussions such as a panel discussion where students interact with invited senior researchers or industry partners. Suggestions such as these will be taken into consideration in the organisation of future SCSs.

## Notes

^a^ www.h3abionet.org

^b^ scsa2017.iscbsc.org

^c^ scsa2017.iscbsc.org/2017-abstracts
